# Mortality forecasting in Colombia from abridged life tables by sex

**DOI:** 10.1186/s41118-018-0038-6

**Published:** 2018-10-01

**Authors:** Gisou Diaz, Ana Debón, Vicent Giner-Bosch

**Affiliations:** 1 Universidad del Tolima, Barrio Santa Helena Parte Alta, 730006299, Ibagué, Tolima, Colombia; 2 Universitat Politècnica de València, Centro de Gestión de la calidad y del cambio, Camino de Vera s/n, Valencia, E-46022 Spain

**Keywords:** Mortality estimation, Lee-Carter model, Mortality forecasting, Life expectancy

## Abstract

**Background:**

An adequate forecasting model of mortality that allows an analysis of different population changes is a topic of interest for countries in demographic transition. Phenomena such as the reduction of mortality, ageing, and the increase in life expectancy are extremely useful in the planning of public policies that seek to promote the economic and social development of countries. To our knowledge, this paper is one of the first to evaluate the performance of mortality forecasting models applied to abridged life tables.

**Objective:**

Select a mortality model that best describes and forecasts the characteristics of mortality in Colombia when only abridged life tables are available.

**Data and method:**

We used Colombian abridged life tables for the period 1973–2005 with data from the Latin American Human Mortality Database. Different mortality models to deal with modeling and forecasting probability of death are presented in this study. For the comparison of mortality models, two criteria were analyzed: graphical residuals analysis and the hold-out method to evaluate the predictive performance of the models, applying different goodness of fit measures.

**Results:**

Only three models did not have convergence problems: Lee-Carter (LC), Lee-Carter with two terms (LC2), and Age-Period-Cohort (APC) models. All models fit better for women, the improvement of LC2 on LC is mostly for central ages for men, and the APC model’s fit is worse than the other two. The analysis of the standardized deviance residuals allows us to deduce that the models that reasonably fit the Colombian mortality data are LC and LC2. The major residuals correspond to children’s ages and later ages for both sexes.

**Conclusion:**

The LC and LC2 models present better goodness of fit, identifying the principal characteristics of mortality for Colombia.

Mortality forecasting from abridged life tables by sex has clear added value for studying differences between developing countries and convergence/divergence of demographic changes.

## Introduction

The study of mortality and its characteristics and forecasting allow us to understand population dynamics and their tendencies. Phenomena such as population growth and the reduction of mortality are of great interest given the economic and social impact they have on the development of countries.

Different models have been developed in recent years to describe mortality (Booth and Tickle 2008; O’hare and Li 2017). Models for the estimation of dynamic life tables are used to graduate the crude death rates and to analyze mortality behavior (Cairns et al. 2011; Andres et al. 2018). The (Lee and Carter 1992) model is one of the best-known and most applied methods in the demographic and actuarial area worldwide. Numerous extensions and modifications of this model have been presented by adding more terms to the original model (among others, Booth et al. (2002), Renshaw and Haberman (2003), Cairns et al. (2009), Haberman (2011)).

This model has been used to study mortality in countries in Central and South America. In Mexico, García-Guerrero and Mellado (2012) and Aburto and García-Guerrero (2015) project mortality using the Lee-Carter model, while Ornelas (2015) fits the Lee-Carter, Renshaw-Haberman, and Age-Period-Cohort (APC) models to obtain fitted rates for the insurance market corrected by general mortality. In Argentina, mortality has been studied by Belliard and Williams (2013), Andreozzi and Blaconá (2011), Andreozzi (2012), and Blaconá and Andreozzi (2014). In this last work, a description of the functional data methodology proposed by Hyndman and Ullah (2007) is presented, which represents an advance over the original Lee-Carter model since it uses nonparametric smoothing to reduce the inherent randomness in the observed data, and the decomposition of the demographic components permits use of classic principal components (Blaconá and Andreozzi 2014). On the other hand, for Chile, Lee and Rofman (1994) extend the Lee-Carter model to solve the problems of incomplete census data. For Costa Rica, Aguilar (2013) uses two variants of the Lee-Carter model for the estimation of life expectancy; the two projections show very similar behavior and reveal higher values than the official ones.

In addition, when we analyze mortality in Latin America, it is important to mention the growth of crime and violent deaths from homicide in some countries in the region. According to Levitt and Rubio (2000), homicide rates in Colombia are among the highest in the world, the homicide rate in Colombia being three times higher than Brazil or Mexico, and ten times higher than Argentina or Uruguay. Garfield and Llanten (2004) analyse the fact that Colombia has the highest level of deaths due to homicide and armed conflict. During the years 1980–2003, many of the deaths were a direct result of armed conflict; others were related to personal vendettas, vigilantism, revenge attacks, easy access to firearms, competition within the illicit drug trade, and the impunity of the law enforcement services.

The application of the Lee-Carter type of model has been little explored for data from Colombia. Reyes (2010) selected a model to make a projection of fiscal spending on pensions for a horizon of 50 years through the study of Colombian mortality for the period 1953–2005. In this paper, the author compares three models for the projection of mortality rates: Lee and Carter (1992) and two variants of this: bms proposed by Booth et al. (2002) and fdm proposed by Hyndman and Ullah (2007), all implemented using the demography (Hyndman et al. 2014) R-package. More recently, Ochoa (2015) presents an application of the Lee-Carter model to estimate Colombia’s mortality for the years 1951–1999, using three different R-packages: demography, ilc (Butt et al. 2014), and gnm (Turner and Firth 2015).

Unlike these authors, in this study, we incorporate more extensions of the Lee-Carter model. Some of them incorporate the cohort effect, a new element for the analysis of mortality in Colombia. Two R-packages were used: gnm and the recent StMoMo (Andres et al. 2018). This package provides preset functions for defining the most common models available in the mortality forecasting literature.

In order to analyze the characteristics of mortality and related demographic phenomena, we made forecasts of mortality that provided several demographic indicators. These were used to describe phenomena such as ageing, demographic transition, standard of living, or inequalities in health (Bertranou 2008). The indicators that are included in mortality studies usually come from population indicators, social indicators or indicators of standard of living, inequality, and poverty (Lora 2008). Among the indicators that relate to mortality and current population trends are life expectancy at birth, life expectancy at age 65, the modal age at death, the Lorenz mortality curve, and the Gini mortality index.

We also think it is appropriate to emphasize the use of abridged life tables in this work. In developed countries (Europe and the USA among others), studies similar to ours mainly use full life tables, whereas for Latin America, this does not happen. Hence, this work can be a benchmark in the use of mortality models with abbreviated tables. In addition, in the database that we use in this paper, the Latin American Human Mortality Database (LAHMD), information is collected through abbreviated life tables in a homogeneous way for the countries according to the available information.

The aim of this paper is to select the best model to forecast the probability of death that represents the characteristics of Colombian mortality when abridged life tables are available. From these results, we made calculations and forecasts of some mortality indicators. The models were adjusted to abridged life tables for Colombia in the period 1973–2005 with data from the Latin American Human Mortality Database (Urdinola and Queiroz 2017). Although this paper only applies graduation and projection to the Colombian abridged life tables, the methodology can be extended to abridged life tables in any geographical area.

The rest of this article is structured as follows. The “Methodology” section describes the fitted models, the criteria for their comparison, and the mortality indicators studied. Then, the “Application to mortality data from Colombia” section presents and discusses the results. The “Conclusion” section ends the paper.

## Methodology

### Mortality models

The Lee and Carter (1992) model expresses the mortality rate (*m*_*xt*_) as a measure that depends on the individual’s age and the corresponding analysis period through an exponential function of these variables.

1 $$ m_{xt}=\exp (a_{x} + b_{x} k_{t}+ \epsilon_{xt})  $$

A modification to this model was proposed by Debón et al. (2008) where the logit transformation is used for probability of death (*q*_*xt*_), as the original Lee-Carter model did not guarantee estimates for *q*_*xt*_ that did not exceed the value 1. The modified Lee-Carter model has the following expression:

2 $$ \text{logit}(q_{xt})= \ln\left(\frac{q_{xt}}{1-q_{xt}}\right)= a_{x} + b_{x} k_{t}+\epsilon_{xt},  $$

where *a*_*x*_ is the age-dependent parameter that describes the overall profile of mortality over age, *b*_*x*_ is the age-dependent sensitivity parameter that represents the change in mortality at age *x* when mortality changes over time, and *k*_*t*_ is the mortality index, a parameter that represents the trend in mortality over time.

The Lee-Carter model with two terms (LC2) represents a particular case of the generalized Booth et al. (2002) model, with an additional bilinear term $b_{x}^{2}k_{t}^{2}$ to modify mortality trends over time. It has been applied to mortality data from European countries such as Spain (Debón et al. 2008) and Italy (Carfora et al. 2017). The expression of LC2 is:

3 $$ \text{logit}(q_{xt})= a_{x} + b_{x}^{1} k_{t}^{1} + b_{x}^{2}k_{t}^{2}+\epsilon_{xt},  $$

where $b_{x}^{2}$ is a second age-dependent parameter representing the change in mortality at age *x* when mortality changes over time, and $k_{t}^{2}$ is a second time-dependent parameter representing the trend in mortality. On the other hand, Richards (2008) highlights the extraordinary importance of the cohort effect, the cohort is defined as the year of birth (*c*=*t*−*x*), in the study of mortality patterns for actuaries. Richards (2008) is a valuable review of the techniques used to identify and model this effect.

Therefore, other models considered in this study include the cohort effect as proposed by Renshaw and Haberman (2006), known as the Lee-Carter model with cohort effect (LCC):

4 $$ \text{logit}(q_{xt})=a_{x} + b_{x}^{1} k_{t} + b_{x}^{2} \gamma_{c} + \epsilon_{xt},  $$

and the model Age-Period-Cohort (APC) (Tabeau 2001) that is obtained when we replace $b_{x}^{1}=1$ and $b_{x}^{2} =1$ in Eq. (4):

5 $$ \text{logit}(q_{xt})=a_{x}+ k_{t} + \gamma_{c} + \epsilon_{xt}.  $$

Model (4) is an extension of the Lee-Carter model (2) where a bilinear term $b_{x}^{2}\gamma _{c}$ is added to indicate a cohort effect that shows the behavior of mortality by year of birth (Renshaw and Haberman 2006). In this case, $b_{x}^{2}$ is an age-dependent sensitivity parameter representing the change in mortality at age *x* in reference to cohort mortality, and *γ*_*c*_ is a parameter representing the trend of mortality across cohorts. When in this model the term $b_{x}^{2}=1$, we obtain the Renshaw-Haberman model (RH). The APC model involves independently analyzing the effect of age, period, and cohort on the probability of death (Currie et al. 2006).

The Cairns-Blake-Dowd (CBD)mortality model suggested by Cairns et al. (2006) proposes a predictor structure with two age-period terms, no static age function, and no cohort effect:

6 $$ \text{logit}(q_{xt})=k_{t}^{1} + (x-\overline{x}) k_{t}^{2} + \epsilon_{xt},  $$

where $\overline {x}$ is the mean age in the data.

Cairns et al. (2009) introduce a generalization of the CBD model where it is suggested that the impact of the cohort effect on a specific cohort decreases over time and therefore is expressed as:

7 $$ \text{logit}(q_{xt})=k_{t}^{1} + (x-\overline{x})k_{t}^{2} + (x_{c} - x)\gamma_{c}+\epsilon_{xt}.  $$

where *x*_*c*_ is a constant parameter to be estimated.

The model is typically known as the M8 model. The expressions of the models described above are summarized in Table 1 with their respective constraints to guarantee the identifiability of the models. The models in Table 1 were fitted with the R-package gnm by Turner and Firth (2015) and StMoMo by Andres et al. (2018), respectively.

**Table 1:** List of mortality models equations and parameter constraints

Mortality model	Formula	Parameter constraints
gnm R-package		
Lee-Carter (LC)	logit(*q*_*xt*_)=*a*_*x*_+*b*_*x*_*k*_*t*_	$\sum _{x} b_{x} = 1$, $k_{t_{0}} = 0$
Lee-Carter with two terms (LC2)	$\text {logit}(q_{xt})=a_{x} + b_{x}^{1} k_{t}^{1} + b_{x}^{2}k_{t}^{2}$	$\sum _{x}b_{x}^{i} = 1, k_{t_{0}}^{i} = 0, i=1,2$
Lee-Carter with cohort (LCC)	$\text {logit}(q_{xt})=a_{x} + b_{x}^{1}k_{t} + b_{x}^{2}\gamma _{c}$	$\sum _{x} b_{x}^{i} = 1$, $k_{t_{0}} = 0$, $\gamma _{c_{0}} = 0$
Age-Period-Cohort(APC)	logit(*q*_*xt*_)=*a*_*x*_+*k*_*t*_+*γ*_*c*_	$k_{t_{0}} = 0$, $\gamma _{c_{0}} = 0$
StMoMo R-package		
Renshaw-Haberman (RH)	$\text {logit}(q_{xt})=a_{x}+b_{x}^{1} k_{t}^{1}+\gamma _{c}$	$\sum _{x} b_{x}^{1}=1,\sum _{t} k_{t}=0$, $\sum _{c} \gamma _{c}=0$
Cairns-Blake-Dowd (CBD)	$\text {logit}(q_{xt})= k_{t}^{1} + (x-\bar {x}) k_{t}^{2}$	no constraints
Generalization of CBD (M8)	$\text {logit}(q_{xt})= k_{t}^{1} +(x - \bar {x})k_{t}^{2}+(x_{c} - x)\gamma _{c}$	$\sum _{c} \gamma _{c}=0$

### Comparison of the models

For the comparison of mortality models, two criteria were analyzed: graphical residuals analysis and the hold-out method to evaluate the predictive performance of the models, applying different goodness of fit measures.

In general, there are three strategies for the validation of the results of the predictions:

1. Evaluate the model in a test sample different to the fitting sample,
2. Develop the model with 75% of the sample and calculate the predictive power with the remaining 25%, or
3. Use the same sample but calculate predictive indicators using bootstrap techniques.

In this paper, we use the second one as we only have one large sample. Specifically, we use the hold-out method, which separates the data into two subsets, one used to train the model and other one to perform the validation test (Blum et al. 1999). We used 75% of the original periods to develop the models (training set) and calculated the predictive power with the remaining 25% of the periods (validation set).

The steps in the hold-out were as follows:

1. Mortality models were fitted to the training dataset.
2. The indexes *k*_*t*_, $k_{t}^{2}$, and *γ*_*c*_ were predicted, using a time series model (ARIMA) for each index in the validation period.
3. Death probability predictions $(\hat {q}_{xt})$ were generated with the predicted indexes (obtained in the previous step) for the validation dataset.
4. The model predictions ($\hat {q}_{xt}$) were compared with the observed mortality probabilities (*q*_*xt*_) in the validation period obtaining measures of goodness of fit.

The measures of goodness of fit used were the root mean square error (RMSE) and mean absolute porcentual error (MAPE), used in a previous paper (Díaz and Debón 2016) and whose expressions are:

8 $$ \text{RMSE}(\hat{q}_{xt})= \sqrt{\sum_{x}\sum_{t} \frac{(q_{xt}- \hat{q}_{xt})^{2}}{n_{x} T}}, \;\; x=1,...,n_{x},\; t=1,...,T  $$

and

9 $$ \text{MAPE}(\hat{q}_{xt})= \frac{\sum_{x}\sum_{t} \frac{|q_{xt}- \hat{q}_{xt}|}{q_{xt}}}{n_{x} T} 100\%, \;\; x=1,...,n_{x},\; t=1,...,T  $$

where *n*_*x*_ is the number of age groups and *T* is the total number of years.

In addition, diagnostic checks on the fitted model by plotting standardized deviance residuals were carried out as sole use of goodness of fit measures is not a satisfactory diagnostic indicator in our experience (Debón et al. 2008; Debón et al. 2012). In the graphical analysis of these residuals, their behavior with respect to age, period, and cohort was evaluated through dispersion plots. This allowed us to analyze the variation of residuals, and we were able to perceive the improvements produced by some models in specific ages and years. Since it is assumed that standardized deviance residuals are independent and identically distributed according to a standard normal distribution of *N*(0,1) in those plots, we should observe that the residuals are randomly distributed. The expression for deviance residuals based on a binomial distribution for the number of deaths is:

$$r_{{dev}_{xt}}=\text{sign}(d_{xt}-\hat{d}_{xt}) \sqrt{2\left[d_{xt} \log\left(\frac{d_{xt}}{\hat{d_{xt}}}\right) + (E_{xt}-d_{xt})\log\left(\frac{E_{xt}-d_{xt}}{E_{xt}-\hat{d}_{xt}}\right)\right]}, $$

where *d*_*xt*_ denotes the observed number of deaths, and *E*_*xt*_ is the number initially exposed to risk at age *x* in year *t*.

The reference interval (− 2.2) for 95.5% of standardized deviance residuals permits the identification of outliers, although sometimes (− 2.5, 2.5) is used to capture 99%.

### Mortality indicators

The analysis of mortality indicators is essential to assess a country’s social, economic, and health status. Within the basic demographic indicators, we find the so-called population indicators that allow us to describe the structural characteristics and behavior of a population. This group includes birth and fertility indicators, age-specific mortality rates, and life expectancy, among others. Another group of mortality indicators summarizes the associations between health inequalities and socioeconomic indicators, such as the modal age at death, the Lorenz mortality curve, and the Gini mortality index (Debón et al. 2012).

- Life expectancy at age *x* (*e*_*x*_):
  Life expectancy represents the average number of years left to live for survivors at age *x* if existing mortality conditions prevail, the expression is:
  10 $$ e_{xt} = \frac{T_{xt}}{l_{xt}},\;\;\;\; t=1,...,T  $$
  where *T*_*xt*_ corresponds to the remaining lifetime for the individuals of a generation from age *x* to its complete extinction and *l*_*xt*_ the number of survivors at the same age *x*.
  In this paper, we obtain life expectancy at birth, *e*_0*t*_, and life expectancy at age 65, *e*_65*t*_, by substituting *x*=0 and *x*=65, respectively, in expression (10). Life expectancy at birth is defined as the average number of years that generation’s newborns in each age group would live under the living conditions observed in a given setting in year *t*. Similarly, life expectancy at 65 years is defined as the average number of years that would be lived from 65 years of age, the components of a generation of individuals in each age group subject to the living conditions observed in a given environment, throughout the year *t*.
- Modal age at death.
  Modal age at death (*M*_*t*_) is an indicator of longevity. It represents the age at which the maximum number of deaths occurs in a population. In a life table, it indicates the age at which most individuals in the initial fictitious cohort die. According to Canudas-Romo (2008), the modal age at death is largely influenced by the mortality rate at more advanced ages and by infant mortality. Thus, the modal age at death may reflect changes in the probability of death that are not detected by life expectancy.
- Lorenz curve of mortality.
  The Lorenz curve, which originated in an economic context, is considered essential for making a diagnosis of the economic situation of a country and its economic and social policy (Lee 1997). It is usually used to represent the distribution of income or welfare among the population. When everyone has the same fraction of total income, we can say that income is distributed equally among members of the population (Lora 2008).
  In the context of this study, we have the Lorenz curve of mortality, which represents the distribution of the age at death of the individuals in a population. To obtain the curve, the proportion of deaths before age *x* are plotted on the x-axis against the cumulative proportion of years that these individuals lived on the ordinate. Then the points are joined up, always leaving a curve below the diagonal. When the number of years lived is divided equally in the whole population, the Lorenz curve coincides with the diagonal. On the other hand, if the number of years lived is concentrated on a single individual it would be represented by the coexistence of the Lorenz curve with the bottom horizontal and the right-hand vertical axis. Llorca et al. (2000).
- Mortality Gini Index.
  According to Singh et al. (2017) the Gini index, which summarizes the Lorenz curve, is considered the most useful measurement to analyze inequality in life expectancy. It is calculated as an additional feature of the table, thus evaluating the inequality between individuals corresponding to the years lived by a person to death. If the mortality Gini index is close to zero, it indicates that all individuals die at approximately the same age; while if it is close to one, it indicates differences in age at death. Therefore, a large number of individuals die at a very early age and very few survive more than the average (Llorca et al. 1998).
  There are different alternatives for the calculation of the Gini index that depend on whether or not the data are grouped. In a complete table, its calculation requires specific mortality probabilities at age *x* (*q*_*x*_), the number of survivors at age *x* (*l*_*x*_), and the total number of years lived from age *x* (*T*_*x*_). The expression of the Gini index at birth in a given year *t* is given in Shkolnikov et al. (2003)
  $$\mathrm{G_{0t}}= \frac{\sum\limits_{x=0}^{\omega-1} (f_{xt}-g_{xt})}{\sum\limits_{x=0}^{\omega-1} f_{xt}}, \;\;\;\; t=1,...,T $$
  where *ω* represents the highest age in the life table, and
  $g_{xt} = \frac {T_{0t}-T_{xt}-{xl}_{xt}}{T_{0t}}$, $f_{xt} = \frac {l_{0t}-l_{xt}}{l_{0t}}$.
  Another expression of the Gini index that is commonly used for abridged life tables is the proposal in Rodríguez (2007), where this indicator of mortality for Colombian data is calculated for the year 2000 for all departments and for Colombia, figures that can be references to assess our calculations. Its expression is as follows:
  11 $$ \mathrm{G_{x_{0}t}}=\left|1-\sum_{i=x_{0}}^{\omega}(N_{it} - N_{(i-1)t})(Y_{(i-1)t} + Y_{it})\right|, \;\;\;\; t=1,...,T  $$
  where $N_{it}= \frac {\sum \limits _{x=x_{0}}^{i}d_{xt}}{\sum \limits _{x=x_{0}}^{\omega }d_{xt}}$ is the cumulative proportion of deaths at age *i*, and $Y_{it} = \frac {\sum \limits _{x=x_{0}}^{i}d_{xt}\bar {x}}{\sum \limits _{x=x_{0}}^{\omega }d_{xt} \bar {x}}$ is the cumulative proportion of the years that these individuals lived, *ω* represents the most advanced age on the life table, $\bar {x}$ is the mean age at death of individuals dying between the exact ages *x* and the following age in the life table, and *d*_*xt*_ is the number of deaths until age *i* in year *t*. For the first age in the life table, $N_{x_{0}-1}=0$ and $Y_{x_{0}-1}=0$. In this paper, we calculate the Gini index at birth, *G*_0*t*_, and the Gini index at age 65, *G*_65*t*_, substituting in expression (11) *x*_0_=0 and *x*_0_=65, respectively.

## Application to mortality data from Colombia

### Data

The data used in this study came from mortality tables constructed for Colombia in the period 1973–2005, using information from the Latin American Human Mortality Database (Urdinola and Queiroz 2017). In this database, the ages are grouped: [0–1], [1–5], [5–10], and the remainder in 5-year age groups up to 85 years. As population data were only available for the last four censuses (1973, 1985, 1993, 2005), the information was completed using linear interpolation to calculate the population between censuses (1974 to 1984, 1986 to 1992, and 1994 to 2004). With these data, it was possible to calculate abridged tables for Colombia from 1973 to 2005. The method of obtaining the mortality tables is described in Díaz and Debón (2016). The following models have been adjusted, taking the age *x* as the midpoint of the above age groups.

### Comparison of the fitted models

**Fig. 1:**
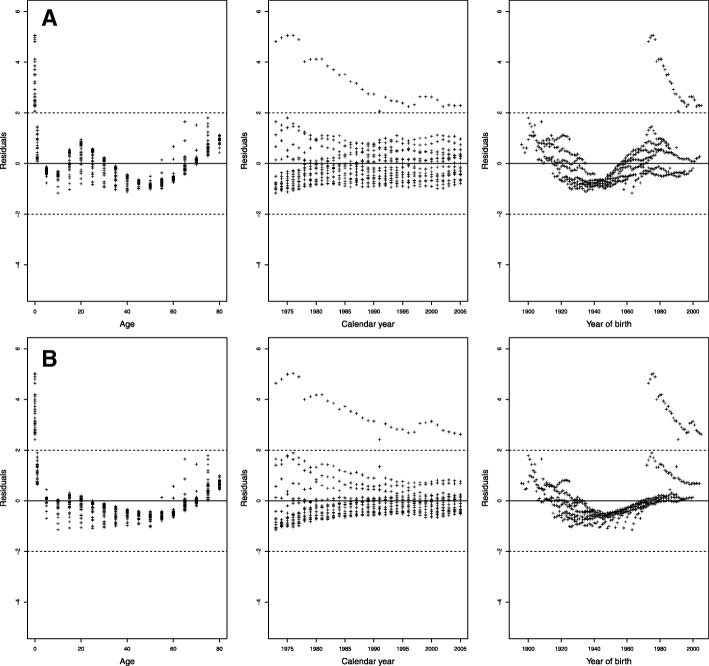
Scatter plots of standardized deviance residuals for the CBD model for the training period 1973–1997. Dashed lines represent interval (−2, 2). **a** CBD for men. **b** CBD for women

The different mortality models in Table 1 were fitted separately for men and women to the data for Colombia, and some of them presented difficulties. The models with cohort effect can present problems of estimation of the parameters, especially when the intervals for the age or the periods are of different amplitudes (Holford 2006). In our study, convergence problems were presented for the LCC model using the gnm R-package and for the RH and M8 models with the StMoMo R-package using the data for men. The convergence problem for mortality models with cohort effect has been pointed out by other authors such as Debón et al. (2010), Hunt and Villegas (2015), and Kennes (2017). On the other hand, the CBD assumes that mortality is linear on the logit scale, so it only works well for advanced ages, causing very high residuals at early ages and poor behavior in general for residuals (see Fig. 1).

Using the hold-out method, an assessment of the fitting and predictive performance of the three mortality models that did not present convergence problems was carried out: LC, LC2, and APC. For these three models, both the fitted and projected values were compared to the probabilities of death observed in each period by the goodness-of-fit measures RMSE and MAPE in expressions (8) and (9), respectively. In fact, we used 75% of the original periods (years 1973–1997) to fit the models (training set) and calculated the predictive power with the remaining 25% (validation set) of the periods (years 1998–2005).

Table 2 shows that according to the calculated goodness of fit measurements in the training period, the LC2 model has the best fit because it has the lowest values of RMSE and MAPE in both sexes. As for the predictive performance of the models evaluated in the validation set, we can say that LC2 has lower MAPE values (the same value 12.63 in both sexes). However, according to RMSE values, LC2 predicts better for men while LC predicts better for women. Regarding the APC model, we can say that it presents high values of RMSE and MAPE for both sexes in the two evaluation periods, so it was discarded for the calculation of mortality indicators and for the graphical evaluation of residuals. Although the APC model has a worse fit, this does not necessarily imply that the cohort effect is not important, but it is difficult to fit with abridged life tables.

**Table 2:** Measures of goodness of fit for the fitted models and mortality indicators

		Training dataset (years 1973–1997)				Validation dataset (years 1998–2005)			
		RMSE		MAPE		RMSE		MAPE	
Model	d.f.	Men	Women	Men	Women	Men	Women	Men	Women
LC	391	0.1149	0.0908	5.89	8.28	0.0061	*0.0050*	15.66	18.70
LC2	352	*0.0753*	*0.0697*	*4.01*	*6.49*	*0.0056*	0.0071	*12.63*	*12.63*
APC	305	0.1237	0.1149	10.08	15.50	0.0174	0.0175	46.60	27.79
LCC	264	–	0.4861	–	76.59	–	0.0171	–	62.00
RH	290	–	0.1001	–	9.87	–	0.3134	–	19.24
M8	299	–	0.1357	–	30.05	–	0.4616	–	49.74
Indicator									
*e*_0*t*_ - LC		0.5582	0.3916	0.68	0.37	0.9160	1.0778	*0.97*	1.41
*e*_0*t*_ - LC2		*0.3921*	*0.0754*	*0.43*	*0.09*	*0.7975*	*0.3178*	1.03	*0.37*
*G*_0*t*_ - LC		0.0054	0.0082	2.23	3.77	0.0122	0.0213	*5.97*	17.09
*G*_0*t*_ - LC2		*0.0048*	*0.0031*	*1.95*	*1.77*	*0.0112*	*0.0135*	6.07	*10.73*
*e*_65*t*_ - LC		0.1503	0.1545	0.91	0.89	0.1119	*0.0823*	0.71	*0.43*
*e*_65*t*_ - LC2		*0.1429*	*0.1218*	*0.84*	*0.72*	*0.0573*	0.1763	*0.32*	0.98
*G*_65*t*_ - LC		0.0003	0.0003	0.60	0.75	*0.0002*	*0.0003*	*0.59*	*0.90*
*G*_65*t*_ - LC2		*0.0002*	*0.0000*	*0.59*	*0.63*	0.0003	0.0004	0.63	0.91

Minimum values in italics

Although the LCC, RH, and M8 models were eliminated from the analysis due to convergence problems for men, some results from these models are shown for women in Table 2. It can be seen that the RMSE and MAPE values for these three models are greater than for the LC and LC2 models in the training and validation dataset.

**Fig. 2:**
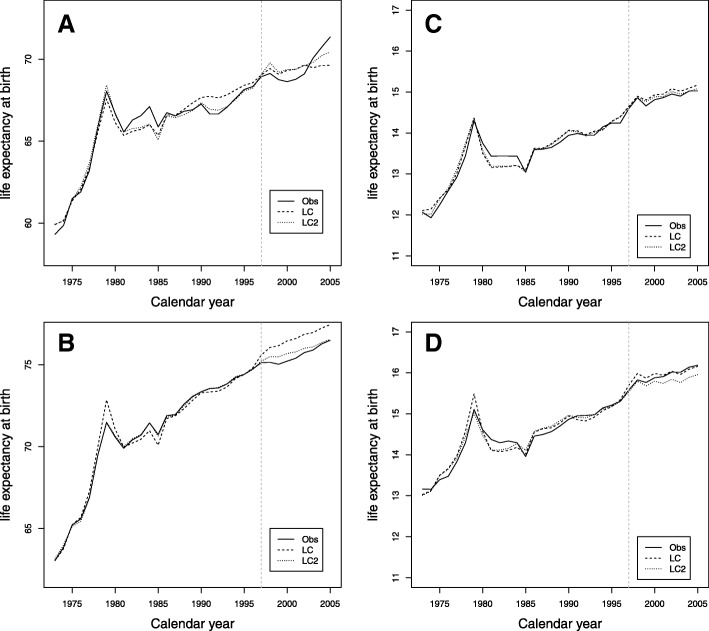
Comparison of life expectancy for the training period 1973–1997 and the validation period 1998–2005. **a** Life expectancy at birth for men. **b** Life expectancy at birth for women. **c** Life expectancy at age 65 for men. **d** Life expectancy at age 65 for women

Figure 2a, b shows the comparison of life expectancy at birth of LC and LC2 for men and women, respectively. For men, in the validation period, the LC2 model presents higher values than the LC model, while the observed data show a more erratic path, rapidly increasing their value in recent years, something that is not captured by the models. For women, in the validation period, LC presents an overestimation of life expectancy at birth while LC2 is close to the observed values. The Fig. 2c, d shows the comparison of life expectancy at age 65 of LC and LC2 for men and women, respectively. The predictions of both models are close to the observed data for men, while for women, LC2 underestimates the values in the validation period.

**Fig. 3:**
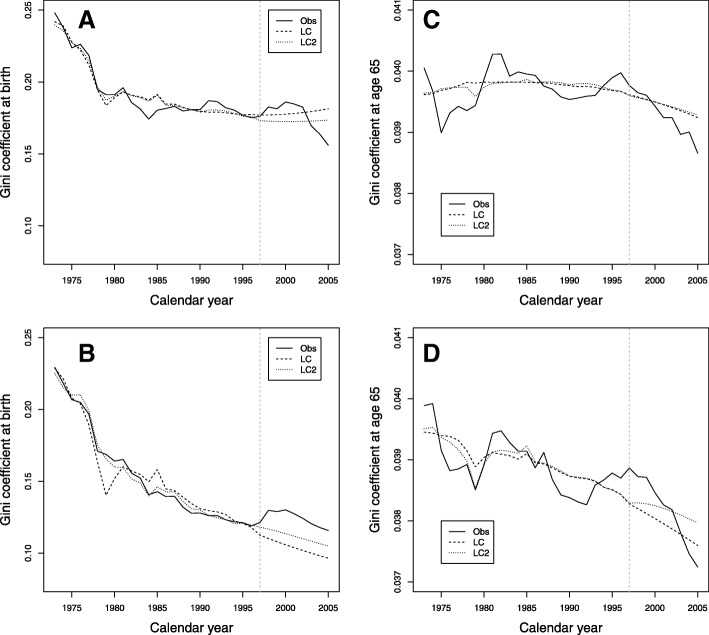
Comparison of Gini index for the training period 1973–1997 and the validation period 1998–2005. **a** Gini index at birth for men. **b** Gini index at birth for women. **c** Gini index at age 65 for men. **d** Gini index at age 65 for women

Figure 3a, b shows the comparison of the Gini index at birth for men and women. For men in the validation period, the models do not capture the decreasing trend present in the observed data. For women, in that period, both models present underestimation, although they show the downward trend present in the observed data. The comparison of the Gini index age 65 of the models for men and women is shown in Fig. 3c, d. For men, in the validation period, both models show an overestimation. For women, in the validation period, the models show the tendency to decrease although they do not capture the rapid drop in the last years present in the observed data.

It was therefore decided to evaluate the effect of fitting and predicting with these two models (LC and LC2) on mortality indicators (see Table 2). In general, LC2 does not improve the predictions in mortality indicators with respect to LC as we can see in Figs. 2 and 3 for life expectancy and the Gini coefficient, especially for age 65.

**Fig. 4:**
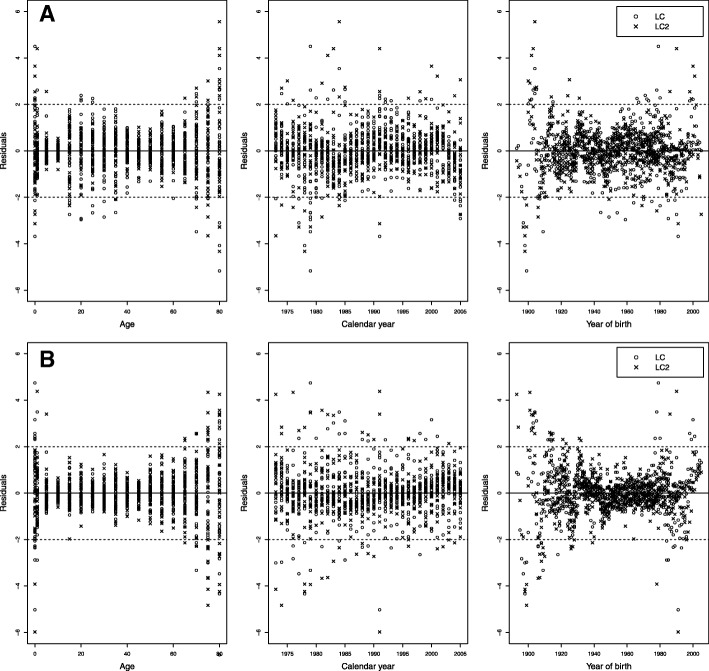
Scatter plots of standardized deviance residuals for the LC and LC2 models at the period 1973–2005 (dashed lines represent the interval (− 2, 2)). **a** Men. **b** Women

Figure 4a, b shows the behavior of the residuals vs. age, period, and cohort for men and women respectively. There is a greater variability in the residuals at infantile ages and advanced ages for both sexes. For men, high values are also perceived for ages between 15 and 40 years, the behavior of LC2 being better than LC only for these ages. In addition, the residuals that depend on the period and cohort present a similar behavior for both models. The analysis of the residuals allows us to state that both models reasonably adjust the data of Colombian mortality.

### Estimation of parameters for the selected models LC and LC2

**Fig. 5:**
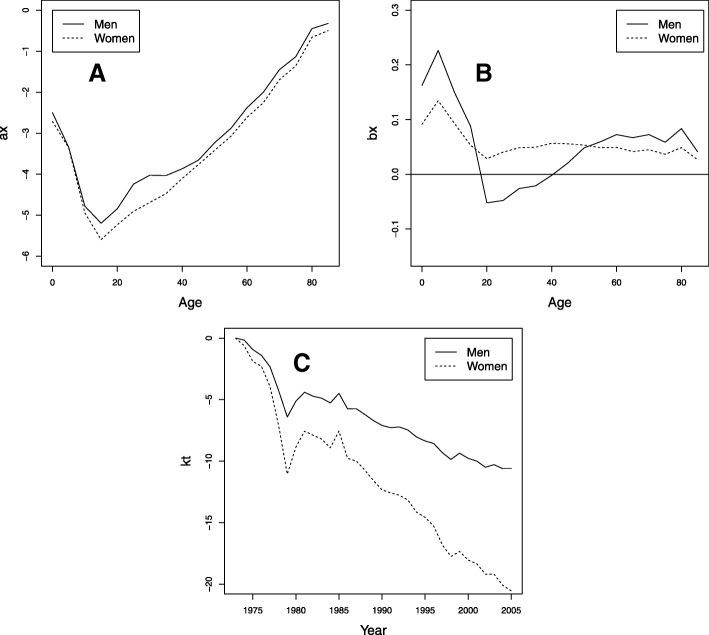
Parameters for the LC model fitted to the Colombian data for the period 1973–2005, men (solid line) and women (dotted line). **a** *a*_*x*_. **b** *b*_*x*_. **c** *k*_*t*_

The first model fitted to Colombian data for the 1973–2005 period was the LC model. Figure 5 presents the parameter estimates of the LC model, which provides different perspectives on mortality behavior and assesses possible differences between the populations of men and women.

Figure 5a shows estimates of *a*_*x*_, where the usual phases of population mortality can be seen. Specifically, the risk of mortality is observed to decrease slowly during the early years of life, with no major differences between men and women. From about 15 years of age, the mortality risk for women begins to be lower than for men, widening this difference between 15 and 39 years of age, and for older ages, the risk of death tends to be similar. This parameter shows the hump phenomenon for mortality, being more marked in men between 15 and 39 years of age. This phenomenon, which is part of the trend of mortality in all countries, known as the young adult mortality hump, is defined as excess mortality in a generally short period of time in young adults. This phenomenon, which has historically been associated with road traffic accidents, in recent years, has been influenced by diseases such as HIV, suicides, and homicides (Remund et al. 2017). In Colombia, the presence of high male mortality among young people is mainly explained by homicides or assaults resulting from violent acts, although they are also related to traffic accidents, Acosta and Romero (2014).

Estimates for the parameter *b*_*x*_ in Fig. 5b indicate how the mortality of each age, *x*, responds to changes in *k*_*t*_, that is, over the years. In women, it takes positive values for all ages, indicating that mortality has decreased for all ages. In men, estimates of this parameter have negative values between the ages of 15 and 39, indicating that mortality increases for these ages over time.

The declining behavior of the *k*_*t*_ index is shown in Fig. 5c. Mortality has decreased in both men and women, and this decrease is much more noticeable in women. The greatest difference between the sexes occurs in the last years of the period analyzed.

**Fig. 6:**
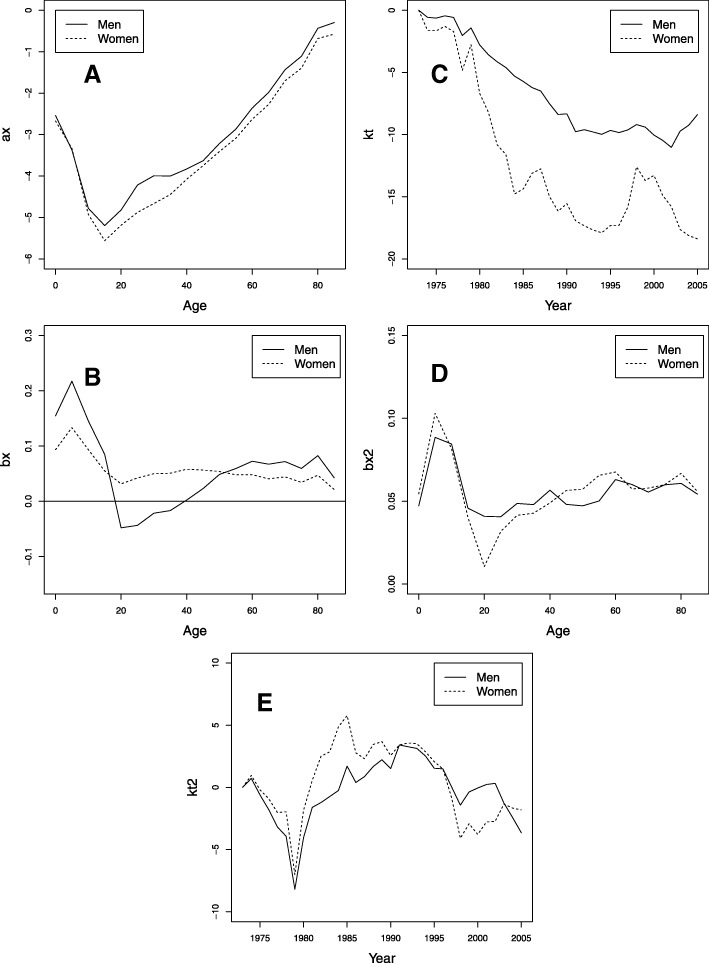
Parameters for the LC2 model fitted to the Colombian data for the period 1973–2005, men (solid line) and women (dotted line). **a** *a*_*x*_. **b** *b*_*x*_. **c** *k*_*t*_. **d**${b_{x}^{2}}$. **e**$k_{t}^{2}$

Figure 6 presents the estimates obtained from the different parameters of the LC2 model. Parameters *a*_*x*_ and *b*_*x*_ have a behavior similar to that observed with the LC model. The general decreasing behavior of the $k_{t}^{1}$ index in Fig. 6c makes the tendency to decrease in mortality evident (similar to the LC model). In women, this trend is more pronounced, especially for a few years following 2000 when men had a slight increase. The differences observed for these years in the behavior of the index $k_{t}^{1}$ in the models LC and LC2 in the men will therefore have very different forecasted values for men.

The behavior of the parameter $b_{x}^{2}$ is shown in Fig. 6d, with higher values in the first years up to 15 years of age and a constant value for the rest of the ages. Figure 6e shows the parameter $k_{t}^{2}$ with values close to zero, although between 1975 and 1980, the values decrease considerably in both sexes. There is also a widespread increase in mortality between the mid-1980s and the end of the 1990s, which was the time of greatest violence in Colombia, and an improvement in mortality in the most recent years. This second term only has an effect for a few years at all ages, improving the fitting of LC for the ages of 20 and 49.

### Calculation and forecasting of mortality indicators

Projections for the *k*_*t*_, $k_{t}^{1}$, and $k_{t}^{2}$ indexes of the LC and LC2 models for the period 2006–2025 were made by fitting an ARIMA model to the whole period 1973–2005, using the respective predicting equation for each case as a projector of the future values of these indexes. Confidence intervals were obtained according to the original proposal of Lee and Carter (1992), that is, from prediction errors in the *k*_*t*_, $k_{t}^{1}$, and $k_{t}^{2}$ indexes projected by the ARIMA models. The auto.arima and forecast functions of the forecast library of R de (Hyndman 2016) were used for the implementation.

**Fig. 7:**
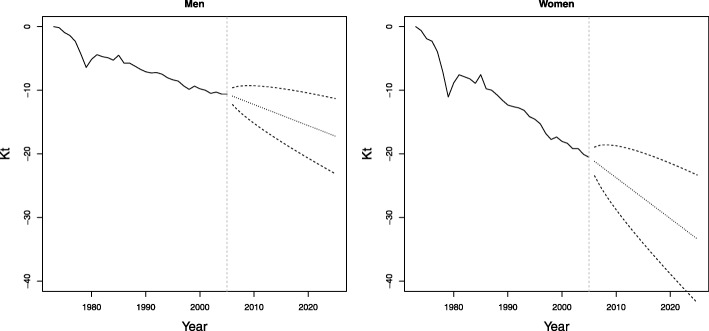
Prediction of the period index of the LC models for the period 2006–2025, Colombia. Dashed lines represent central forecasts, and dotted lines represent 95% prediction intervals

Figure 7 shows the results of the predictions of the *k*_*t*_ index using the LC model for men and women, with their confidence intervals. A trend is projected to continue with decreasing mortality for both men and women, although in women, this projection has a more marked reduction.

**Fig. 8:**
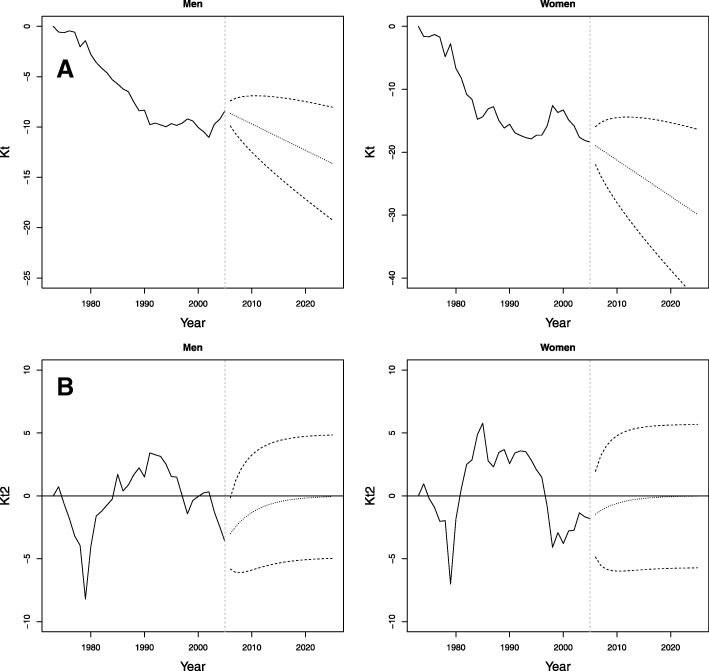
Prediction of the period indexes of the LC2 models for the period 2006–2025, Colombia. Dashed lines represent central predictions, and dotted lines represent 95% prediction intervals. **a** *k*_*t*_. **b**$k_{t}^{2}$

The results of the predictions of the $k_{t}^{1}$ and $k_{t}^{2}$ indexes of the LC2 model with their confidence intervals are shown in Fig. 8a, b, respectively. For the $k_{t}^{1}$ index, although their values increased in recent years for men, according to the adjusted ARIMA(0,1,0), there is a tendency to decrease. The $k_{t}^{2}$ index tends to zero in both sexes (ARIMA(1,0,0)). In this way, forecasted values indicate a tendency for mortality to decrease for both men and women.

**Fig. 9:**
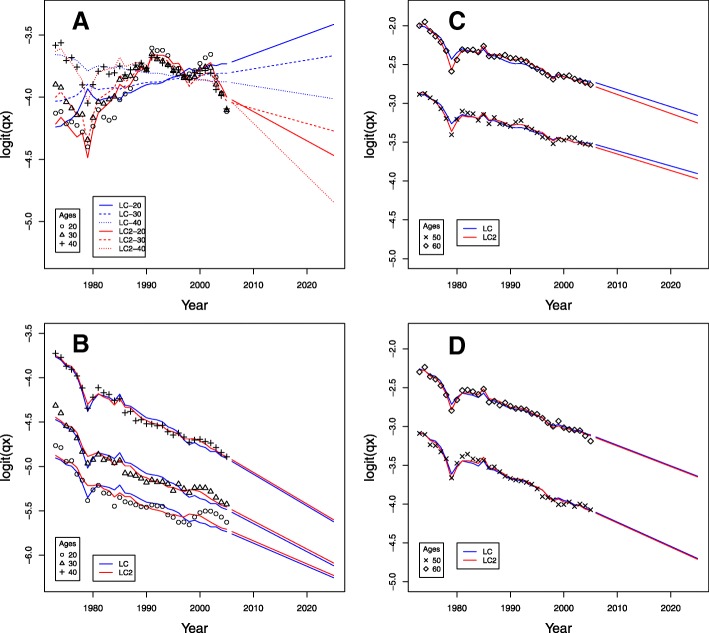
Death probabilities between 1973 and 2005 and predictions up to 2025 for some ages, Colombia. **a** Ages 20–40 years, men. **b** Ages 20–40 years, women. **c** Ages 50–60 years, men. **d** Ages 50–60 years, women

The forecasted probabilities of death for ages 20, 30, and 40 are shown in Fig. 9a, b while ages 50 and 60 years are shown in Fig. 9c, d for men and women, respectively. For men, according to Fig. 9a, the fitted death probabilities for ages 20 and 30 show great differences between the models. The forecasted values for the model LC2 show a decrease in the probabilities of death as we mentioned before. In addition, it was confirmed that the model LC2 fits and predicts better for men, indicating that the inclusion of the second term better adapts the model to changes in trends for intermediate ages. Figure 9b shows the predictions of the probabilities of death for women with a clear downward trend that is more subdued by the age of 20 years. For older ages, 50 and 60 years, there are almost no differences in the fitting and forecasting of the models (see Fig. 9c, d).

Some mortality indicators such as life expectancy at birth and the Gini index were calculated for the period analyzed, and projections were made up to 2025. This was in order to analyze the trends in the coming years and their relationship with demographic changes that are occurring in Colombia.

**Fig. 10:**
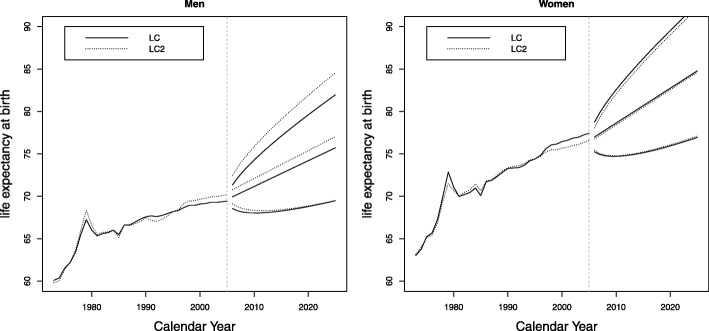
Evolution for life expectancy between 1973 and 2025, Colombia, men and women

The forecasted life expectancy for the Colombian population increased for both sexes for the period 1973–2025 (Fig. 10 in Appendix). For men, the increase was about 10 years and for women 13 years during the period studied, 1973–2005. Furthermore, we can say that life expectancy will increase for both during the forecasted period 2006–2025. Men will have an increase of 7 years from 71 years, and women will experience an increase of 8 years from 76 years. Women will have a higher life expectancy than men (6 years more), thus maintaining the tendency to live longer.

**Fig. 11:**
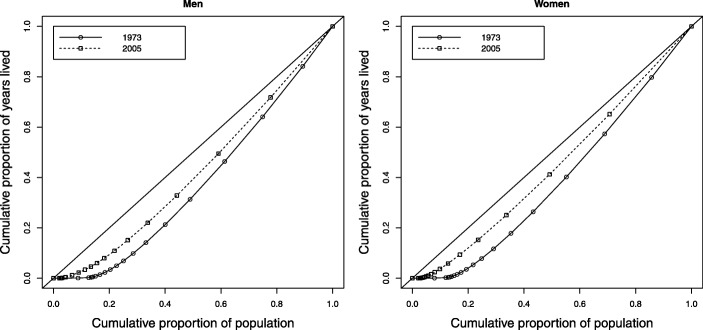
Evolution of Lorenz curve, Colombia, selected years 1973 (solid line) and 2005 (dotted line), men and women

In both sexes, there is a slight tendency towards the diagonal of the Lorenz curves for 2005, with this being the most notable for women. In addition, it can be seen that young children and young people have a small contribution to make in the distribution of the years they live, which shows an inequality in the age at death (or life expectancy) of the Colombian population (Fig. 11 in the Appendix).

**Fig. 12:**
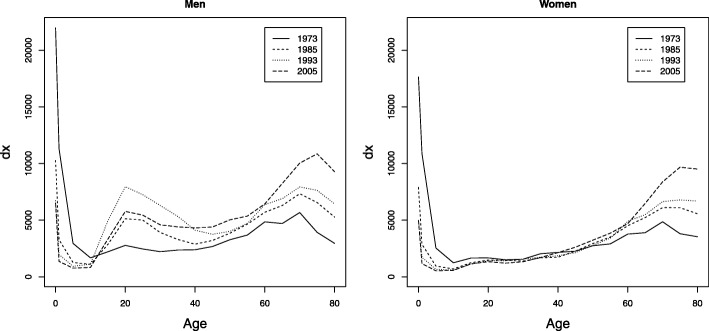
Evolution of the death curve, Colombia, census years, men and women

**Fig. 13:**
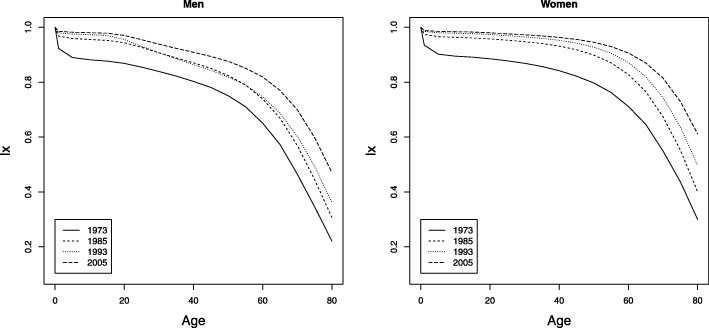
Evolution of the survival curve, Colombia, census years, men and women

To complement the above, the curves of deaths (Fig. 12 in Appendix) and the curves of number of survivors (Fig. 13 in Appendix) are shown for the census years (1973, 1985, 1993, and 2005). The main feature is the increase in age of death for adult ages according to Fig. 12a, b in the Appendix. The mortality hump for young adults showed a significant increase in the last three censuses. The phenomenon of the rectangularization in the survival curve, which implies a displacement of the survivor curve to the upper right corner is shown in Fig. 13a, b in the Appendix. This phenomenon is seen more clearly for Colombian women.

**Fig. 14:**
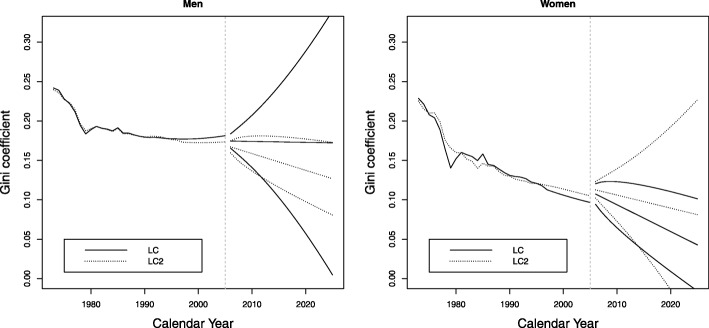
Evolution of the Gini index between 1973 and 2025, Colombia, men and women

The behavior of the Gini index, which decreases for both sexes during the period analyzed, with the decrease being much more marked for women is shown in Fig. 14 in the Appendix. Values decreased for men from 0.24 in 1973 to 0.17 in 2005, and for women from 0.22 to 0.11. Therefore, it can be seen that inequalities in the age at death are greater for men than for women during the entire period analyzed, and the projection is that this trend will continue until 2025. The results found are in accordance with the reported value of 0.11 for Colombia in 2000 in Rodríguez (2007), and it is consistent with the process of improving the country’s quality of life and health.

Considering the modal age at death, we can say that for men during the period 1973 to 1989, the interval was [75, 80] years, and between 1990 and 2005, the modal age at death increased to the interval [80, 85] years. For women, the modal age at death was in the interval [75, 80] years for the period 1973 to 1983, while in the period 1984 to 2005, the modal age of death increased to the interval [80, 85] years. This reinforces the idea that women have been living longer in Colombia for many more years.

## Conclusion

Estimation of mortality from a good forecasting model is important considering the impact that its results have on the different processes of social and economic planning of a country. In some developing countries, data are usually given in age groups because of systematic fluctuations caused by age heaping. This is a phenomenon usual to vital registrations related to age misstatements, usually preferences for ages ending in multiples of five and some other registration difficulties. Therefore, a question of interest in the demographic and actuarial fields is the estimation and forecasting mortality pattern using abridged life tables.

In this paper, we make forecasts of mortality in Colombia that show the behavior of mortality for abridged life tables. Unlike previous studies for Colombia and other Latin American countries, we used a wide variety of extensions of the model Lee-Carter which allowed us to select the model with the best goodness of fit and from this make forecasts of the mortality and estimation of some indicators. It is important to point out that as far as we know, the StMoMo R-package has not to date been used for the graduation of Colombian mortality data and that R-package and gnm allowed us to fit a variety of Lee-Carter extensions.

As in many other countries all over the world, all the models predict better mortality for women as mortality experience for women has less fluctuations. In addition, it is important to highlight the use of these seven models in abridged life tables and the results found despite the non-convergence of some models. In this study, the models presented problems of convergence with the cohort effect with both R-packages for men, except the APC model. The convergence problem for mortality models with cohort effect has been pointed out by other authors such as Debón et al. (2010), Hunt and Villegas (2015), and Kennes (2017). In this study, we would like to remark that the cohort effect presents problems of estimation of the parameters on abridged life tables as cohorts represent subsets of five cohorts with different numbers of observations. On the other hand, the CBD model demonstrated very bad behavior for infants and advanced ages. Therefore, the comparison was carried out by fitting LC, LC2, and APC. In summary, we can conclude that the LC2 model provides a better fit for both sexes, although the improvement of LC2 on LC is mostly for intermediate ages.

Some mortality characteristics were identified for Colombia through the fitting of the LC and LC2 mortality models. The usual behavior of probability of deaths with age: high mortality at infant ages gradually decreases until age 15 and then increases as the population ages. Mortality decreased significantly in the period 1973 to 2005 for most of the ages with a small tendency to increase in recent years for men. The hump phenomenon is observed for mortality mainly in men from 15–39 years which is clearly visualized by the LC model but is discomposed into two terms for the LC2 model. This over-mortality is mainly explained by homicides or assaults resulting from violent acts, although they are also related to traffic accidents. This mortality pattern is more notable in Colombia than in other Latin countries. According to our results, forecasted death probabilities are more feasible with LC2 than with LC especially for men. However, data over a more recent period might still need to be analyzed in order to derive parameter estimates that give reasonable forecasting at all ages.

Phenomena such as over-mortality in young men (hump phenomenon) that mean that the behavior of mortality is different between the sexes are important for insurance companies. Life tables are the tool that the insurance companies use to calculate risk and to value the products that they issue on the market. In Colombia, insurance companies do not bear the phenomenon of the hump in young men in mind for two reasons: firstly, to prevent people from making the decision to postpone the purchase of insurance until completing a certain age to save money, a fact that could mean that these people remain uninsured for many years, and secondly, to avoid obtaining negative values when calculating the value of the reserve to be established by the insurance companies. Applying this measure in the pricing can be important for countries with similar developmental conditions to Colombia in order to prevent the effect of this phenomenon in the pricing.

The forecasting of demographic and mortality indicators allows us to conclude that the Colombian population is immersed in a phenomenon of gradual improvement in its living conditions. Life expectancy remains the most familiar measure of longevity among demographers, and although it reflects the changes in mortality with time, it does it in a smooth way due to its robustness. This is the reason why in the present paper, other indicators were studied: modal age at death, Lorenz curve, and Gini index. The evolution of the modal age at death, the Lorenz curve, and Gini index also confirmed demographic changes in Colombia. Greater longevity in women than in men is confirmed, showing higher life expectancy and a lower Gini index. Therefore, we can conclude that LC2 does not improve the predictions for mortality indicators with respect to LC for life expectancy and the Gini coefficient especially at age 65, although LC2 is better for probabilities forecasting. It can be appreciated that LC is quite poor in terms of prediction, particularly in the age class 20–40 years.

The differences that we observed in the decrease of mortality and the increase in life expectancy between sexes, should be borne in mind by Colombian insurance companies for the production of life tables and the calculation of their products. According to resolution 1555 of 2010 of the Financial Superintendence of Colombia, the life tables that the administrative entities of the General System of Pensions, the General System of Professional Risks, and the life insurance companies use for the production of their products and for actuarial calculations are discriminated by sex. Something different happens in the European Union where according to the board 2004/113/EC of the Court of Justice of the European Union (EU), sex discrimination cannot be established in the goods and services that involve the use of tables of unisex mortality in the insurance sector.

Finally, we would like to point out that although this paper only applies graduation to the Colombian abridged life tables, the methodology can be extended to abridged life tables in any geographical area.

## Appendix

## Abbreviations

LC: Lee-Carter
LC2: Lee-Carter with two terms
APC: Age-Period-Cohort
CBD: Cairns-Blake-Dowd
RMSE: Root mean square error
MAPE: Mean absolute porcentual error
